# Association between the ZJU index and carotid atherosclerosis in patients with type 2 diabetes: A cross-sectional study

**DOI:** 10.1097/MD.0000000000047442

**Published:** 2026-01-30

**Authors:** Xifeng Sun, Xiao Yu, Jiaming Shen, Mengmeng Wang, Yazhao Sun

**Affiliations:** a Department of Cerebrovascular Interventional Neurology, Cangzhou People’s Hospital, Cangzhou, China; b Department of Cardiology, Cangzhou People’s Hospital, Cangzhou, China.

**Keywords:** carotid atherosclerosis, diabetes, insulin resistance, ZJU index

## Abstract

The ZJU index, as an emerging metabolic health indicator, has not yet been clearly associated with atherosclerosis. This study, using carotid atherosclerosis (CAS) as a measure of atherosclerosis, aimed to explore the value of the ZJU index in assessing the relationship between the ZJU index and CAS in patients with type 2 diabetes (T2DM). We employed multivariable-adjusted logistic regression, restricted cubic spline regression, and sensitivity analysis to explore the relationship between the ZJU index and CAS. The predictive ability of the ZJU index for CAS was evaluated using receiver operating characteristic curves. We observed 260 cases of CAS. In the fully adjusted model, the ZJU index was associated with the occurrence of CAS (OR = 1.06, 95% CI: 1.02–1.10, *P* = .007). In the highest ZJU index tertile, the odds of developing CAS were 2.26 times higher compared to those in the lowest tertile (OR = 2.26, 95% CI: 1.32–3.93, *P *= .003). Based on restricted cubic spline analysis, a linear relationship between the ZJU index and CAS was observed in patients with T2DM. receiver operating characteristic analysis showed that the ZJU index has a certain predictive ability for CAS. The ZJU index may serve as a potential predictive indicator of CAS in patients with T2DM.

## 1. Introduction

Type 2 diabetes (T2DM) is one of the most prevalent chronic metabolic diseases worldwide, with its incidence continuing to rise annually, primarily driven by the increase in unhealthy lifestyle habits. One of the primary pathological features of T2DM is metabolic dysfunction, particularly insulin resistance and β-cell dysfunction. The complexity of this disease is reflected not only in its pathophysiological mechanisms but also in its strong association with various other health issues, such as cardiovascular disease.^[1]^ Atherosclerosis is the major pathological basis for the development of cardiovascular disease, and the risk of atherosclerosis is significantly increased in patients with T2DM due to metabolic dysfunction and the presence of insulin resistance.^[2]^

Carotid atherosclerosis (CAS), a manifestation of systemic atherosclerosis in the carotid arteries, is an important indicator for assessing cardiovascular health. The prevalence of CAS among individuals aged 30 to 79 years in China is 27.22%.^[3]^ According to 2020 data, the prevalence of carotid plaques in the global population aged 30 to 79 years is approximately 21.1%, while the prevalence of carotid intima-media thickness (cIMT) is around 27.6%.^[4]^ Therefore, early identification of high-risk groups among patients with T2DM and effective interventions to control the progression of atherosclerosis are crucial strategies for preventing cardiovascular disease.

Insulin resistance promotes the occurrence of atherosclerosis by causing endothelial dysfunction and oxidative stress and has become an important predictor of atherosclerosis.^[5]^ The ZJU index, proposed by Wang et al in a cross-sectional study of 9602 Chinese individuals who underwent liver ultrasound diagnosis, integrates multiple indicators, including fasting blood glucose (FBG), triglycerides (TG), body mass index (BMI), aspartate aminotransferase (AST), and alanine aminotransferase (ALT) levels.^[6]^ Previous studies have shown that the ZJU index is closely related to lipid metabolism, insulin resistance, and obesity.^[7]^ Compared to individual biochemical markers, the ZJU index provides a more comprehensive assessment, effectively reflecting early changes in glucose, lipids, and other metabolic disorders in patients with diabetes. A cohort study conducted by Wu et al demonstrated a positive correlation between the ZJU index and the incidence of diabetes in the general population.^[8]^ However, whether the ZJU index can serve as an effective surrogate marker for atherosclerosis remains unclear. Therefore, this study aims to comprehensively evaluate the relationship between the ZJU index and CAS in patients with T2DM and further explore its potential application in the early screening of metabolic disorders and cardiovascular risk assessment.

## 2. Materials and methods

### 2.1. Study participants

This study consecutively recruited patients aged ≥45 years with T2DM who were hospitalized at Cangzhou People’s Hospital between December 2019 and January 2022. Participants were excluded if they met any of the following criteria: lack of carotid imaging data; suspected familial hypertriglyceridemia; missing data on BMI, TG, ALT, AST, FBG, or other relevant variables; or incomplete clinical information. Ultimately, 896 patients with available ZJU index and carotid imaging data were included in this analysis.

The study was approved by the Ethics Committee of Cangzhou People’s Hospital and the Institutional Review Board (K2024-094-01). All patients provided written informed consent for the review of their medical records and for participation in the study. The research was conducted in accordance with the ethical standards of the Declaration of Helsinki, ensuring that the rights, safety, and well-being of all participants were protected throughout the study.

### 2.2. Data collection

Data on sociodemographic characteristics, including age, gender, BMI, waist circumference, visceral fat area, lifestyle risk factors (such as current smoking), and self-reported medical history (e.g., coronary artery disease and hypertension) were collected. BMI was calculated by dividing the weight (kg) by the height squared (m^2^).

Venous blood samples were collected from the antecubital vein in the morning after a fasting period of at least 8 hours within 24 hours of admission. The tests included the following biochemical parameters: white blood cell count (WBC), hemoglobin (HGB), platelet count (PLT), FBG, hemoglobin A1c (HbA1c), AST, ALT, uric acid, serum creatinine (SCr), total cholesterol (TC), TG, high-density lipoprotein cholesterol (HDL-C), and low-density lipoprotein cholesterol (LDL-C).

The ZJU index was calculated as BMI (kg/m^2^) + FBG (mmol/L) + TG (mmol/L) + 3 × ALT/AST ratio, with an additional 2 for females.^[6]^ The unit conversions are as follows: FBG, mg/dL = mmol/L × 18; TC, LDL-C, and HDL-C, mg/dL = mmol/L × 38.67; TG, mg/dL = mmol/L × 88.57.

### 2.3. Carotid artery measurements

Carotid ultrasound examinations were performed by 2 certified ultrasonographers, all of whom underwent standardized training prior to the study to ensure consistency in measurements. During the examination, the ultrasonographers were blinded to the participants’ baseline characteristics and laboratory results. All measurements were conducted using the same high-resolution ultrasound system (Toshiba Aplio 300, Toshiba Co. Ltd., Tokyo, Japan), with patients positioned in the supine position. The ultrasonographers examined both sides of the common carotid, internal carotid, external carotid, subclavian, and vertebral arteries. cIMT was measured using a linear sensor with a frequency of 10.0 MHz. According to the Mannheim Consensus, the region of interest for cIMT measurement was the distal common carotid artery, located on both sides of the carotid bifurcation.^[9]^ An increase in cIMT was defined as a left/right carotid intima-media thickness ≥1.0 mm. Both average and maximum cIMT were analyzed: average cIMT was defined as the mean value of both common carotid arteries, while maximum cIMT was defined as the larger value of the left or right common carotid artery.^[10]^ Carotid plaques were defined as an intima-media thickness ≥1.5 mm, a focal structure protruding into the arterial lumen by at least 0.5 mm, or >50% of the surrounding intima-media thickness.^[11]^ Carotid stenosis was defined as a narrowing of 50% or more.^[12]^ Patients with any increase in cIMT, plaque formation, or carotid stenosis were diagnosed with CAS. To ensure the reliability of the results, repeat measurements were performed on a subset of patients, and the inter-observer consistency was evaluated. The results showed high consistency between the ultrasonographers’ measurements, effectively minimizing the impact of observational variability on the study outcomes.

### 2.4. Statistical analyses

This study is a cross-sectional analysis, and the sample size was based on available data. A post hoc power analysis was conducted, which showed that the 896 participants (636 in the CAS group and 260 in the Non-CAS group) provide 0.80 statistical power to detect an effect size of 0.16 (Cohen d). Therefore, the sample size is sufficient to support the statistical analysis in this study.

To compare the differences between the CAS group and the Non-CAS group, the normality of all continuous variables was first assessed using the Kolmogorov-Smirnov test. The results showed that all continuous variables were non-normally distributed and were presented as median (Q1, Q3). Therefore, the Mann–Whitney U test was used for group comparisons. Categorical variables were expressed as n (%) and compared using the Chi-square test.

To investigate the relationship between the ZJU index and CAS, relevant covariates with a *P*-value <.05 from the baseline characteristics were incorporated into a logistic regression model. A collinearity check was first performed, and variables with a variance inflation factor >5, such as FBG and TG, were excluded. We used 2 logistic regression models to assess the relationship between the ZJU index and CAS and calculated the odds ratios and 95% confidence intervals. Model 1 was unadjusted, while Model 2 was adjusted for BMI, visceral fat area, WBC, HbA1c, TC, and HDL-C. A restricted cubic spline analysis based on a logistic regression model was conducted to explore the dose-response relationship between the ZJU index and CAS. Receiver operating characteristic curves were used to assess the predictive ability of the ZJU index. Additionally, sensitivity analysis was performed. All statistical analyses were conducted using R software (version 4.4.3). A 2-sided *P*-value of <.05 was considered statistically significant in this study.

## 3. Results

### 3.1. Participant characteristics

Among the participants, 636 were classified into the CAS group. The baseline characteristics of the study population stratified by Non-CAS and CAS are summarized in Table 1. The mean age of the participants was 56 years, with 55.7% being male. Compared to the Non-CAS group, the CAS group exhibited significantly higher BMI, visceral fat area, WBC, FBG, HbA1c, TC, TG, and ZJU index. In contrast, the HDL-C level was significantly lower in the CAS group. These baseline differences provide the clinical context for the subsequent analysis of the association between the ZJU index and CAS, helping to further elucidate the potential role of relevant biomarkers in the pathogenesis of CAS.

**Table 1:** Baseline characteristics of participants stratified by CAS status.

Variables	Total (n = 896)	Non-CAS (n = 260)	CAS (n = 636)	*P*-value
Age, years, Median (Q1, Q3)	56 (51, 64.25)	55 (51, 64)	57 (52, 65)	.213
Male, n (%)	499 (55.7)	146 (56.2)	353 (55.5)	.917
Coronary artery disease, n (%)	184 (20.5)	62 (23.8)	122 (19.2)	.140
Hypertension, n (%)	474 (52.9)	140 (53.8)	334 (52.5)	.773
Current Smokers, n (%)	277 (30.9)	89 (34.2)	188 (29.6)	.196
BMI, kg/m^2^, Median (Q1, Q3)	26.69 (24.58, 29.13)	25.7 (23.61, 27.91)	27.08 (25.02, 29.34)	<.001
WC, cm, Median (Q1, Q3)	86 (83.75, 94)	86 (84, 92.25)	86 (83, 94)	.580
Visceral fat area, cm^2^, Median (Q1, Q3)	109.8 (87, 142)	97.6 (81.15, 130.7)	115.55 (90.38, 146.43)	<.001
WBC, 10^9^/L, Median (Q1, Q3)	6.1 (5.18, 7.27)	5.78 (4.86, 7.03)	6.16 (5.3, 7.33)	.003
HGB, g/L, Median (Q1, Q3)	131 (115, 145)	130 (111, 142)	131 (117, 146)	.050
PLT, 10^9^/L, Median (Q1, Q3)	226 (179, 277)	227.5 (178, 277)	226 (179, 277)	.794
FBG, mg/dL, Median (Q1, Q3)	156.05 (125.06, 207.59)	138.57 (117.85, 173.12)	161.37 (129.7, 215.38)	<.001
HbA1c, %, Median (Q1, Q3)	8.3 (7.1, 9.9)	7.7 (6.9, 9.2)	8.6 (7.27, 10.1)	<.001
ALT, U/L, Median (Q1, Q3)	18 (13.3, 28)	18 (13.3, 25)	19 (13.75, 29)	.119
AST, U/L, Median (Q1, Q3)	17 (14, 22.02)	17 (14, 22)	17 (14, 23)	.932
SCr, μmol/L, Median (Q1, Q3)	62 (52, 72)	62 (52, 71.25)	62 (52, 73)	.504
UA, μmol/L, Median (Q1, Q3)	285 (230, 342)	280.5 (215.75, 339)	287 (238, 343)	.076
TC, mg/dL, Median (Q1, Q3)	182.14 (153.91, 211.62)	175.56 (148.11, 207.37)	185.81 (157.77, 213.17)	.014
TG, mg/dL, Median (Q1, Q3)	133.79 (93.03, 207.32)	116.51 (85.5, 183.84)	144.42 (98.35, 212.86)	<.001
HDL-C, mg/dL, Median (Q1, Q3)	42.54 (35.58, 51.14)	43.7 (36.35, 52.3)	41.76 (34.8, 51.04)	.043
LDL-C, mg/dL, Median (Q1, Q3)	106.42 (79.33, 133.13)	104.88 (77.98, 131.68)	107.39 (79.72, 133.22)	.445
ZJU index, Median (Q1, Q3)	42.17 (38.74, 46.64)	40.22 (36.7, 44.35)	43.08 (39.71, 47.54)	<.001

ALT = alanine aminotransferase, AST = aspartate aminotransferase, BMI = body mass index, FBG = fasting blood glucose, HbA1c = hemoglobin A1c, HDL-C = high-density lipoprotein cholesterol, HGB = hemoglobin, LDL-C = low-density lipoprotein cholesterol, PLT = platelet count, SCr = serum creatinine, TC = total cholesterol, TG = triglyceride, UA = uric acid, WBC = white blood cell count, WC = waist circumference.

### 3.2. The association between the ZJU index and CAS

To investigate the relationship between the ZJU index and CAS, relevant covariates between the Non-CAS and CAS groups were incorporated into a logistic regression model. A collinearity check was first performed, and variables with a VFI >5, such as FBG and TG, were excluded. Subsequently, univariate and multivariate logistic regression analyses revealed a significant association between the ZJU index and the increased likelihood of developing CAS (Table 2).

**Table 2:** Univariate and multivariate logistic regression analysis for CAS.

	Model 1 OR (95% CI), *P* value	*P* for trend	Model 2 OR (95% CI), *P* value	*P* for trend
CAS	–	<.001	–	<.001
ZJU index (per SD increase)	1.11 (1.08–1.14), <0.001	–	1.06 (1.02–1.10), 0.007	–
ZJU index group				
Quartile 1: ≤40.04)	1.0 (Ref)	–	1.0 (Ref)	–
Quartile 2: 40.04–45.03	1.78 (1.27–2.50), <0.001	–	1.26 (0.85–1.85), 0.246	–
Quartile 3: ≥45.03	4.64 (3.14–6.98), <0.001	–	2.26 (1.32–3.93), 0.003	–

Model 1 adjusted for none; Model 2 adjusted for BMI, visceral fat area, WBC, HbA1c, TC, and HDL-C.

BMI = body mass index, CAS = carotid atherosclerosis, CI = confidence interval, HbA1c = hemoglobin A1c, HDL-C = high-density lipoprotein cholesterol, OR = odds ratios, TC = total cholesterol, WBC = white blood cell count.

After adjusting for known confounders, each standard deviation increase in the ZJU index was associated with a 6% increased odds of developing CAS (OR = 1.06, 95% CI: 1.02–1.10, *P* = .007). Further quartile analysis revealed that, in the highest ZJU index tertile, the odds of developing CAS were 2.26 times higher compared to those in the lowest tertile (OR = 2.26, 95% CI: 1.32–3.93, *P* = .003).

**Figure 1.**
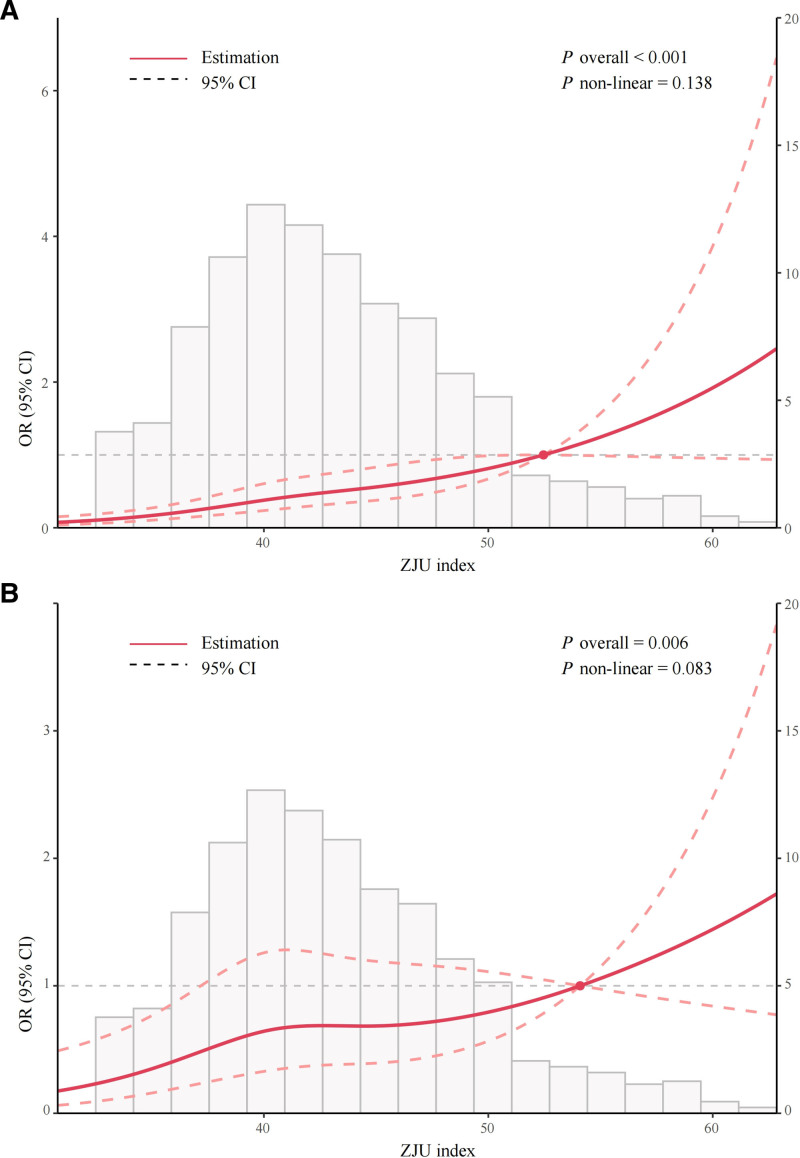
Distribution and restricted cubic spline curves for the ZJU index and the incidence of CAS. (A) Unadjusted model; (B) Model 2 adjusted for BMI, visceral fat area, WBC, HbA1c, TC, and HDL-C. BMI = body mass index, CAS = carotid atherosclerosis, CI = confidence interval, HbA1c = hemoglobin A1c, HDL-C = high-density lipoprotein cholesterol, OR = odds ratios, TC = total cholesterol, WBC = white blood cell count.

The relationship between the ZJU index and CAS was explored using restricted cubic spline analysis. A linear relationship between the ZJU index and CAS was observed (Fig. 1).

### 3.3. Predictive ability of the ZJU index for CAS

**Figure 2.**
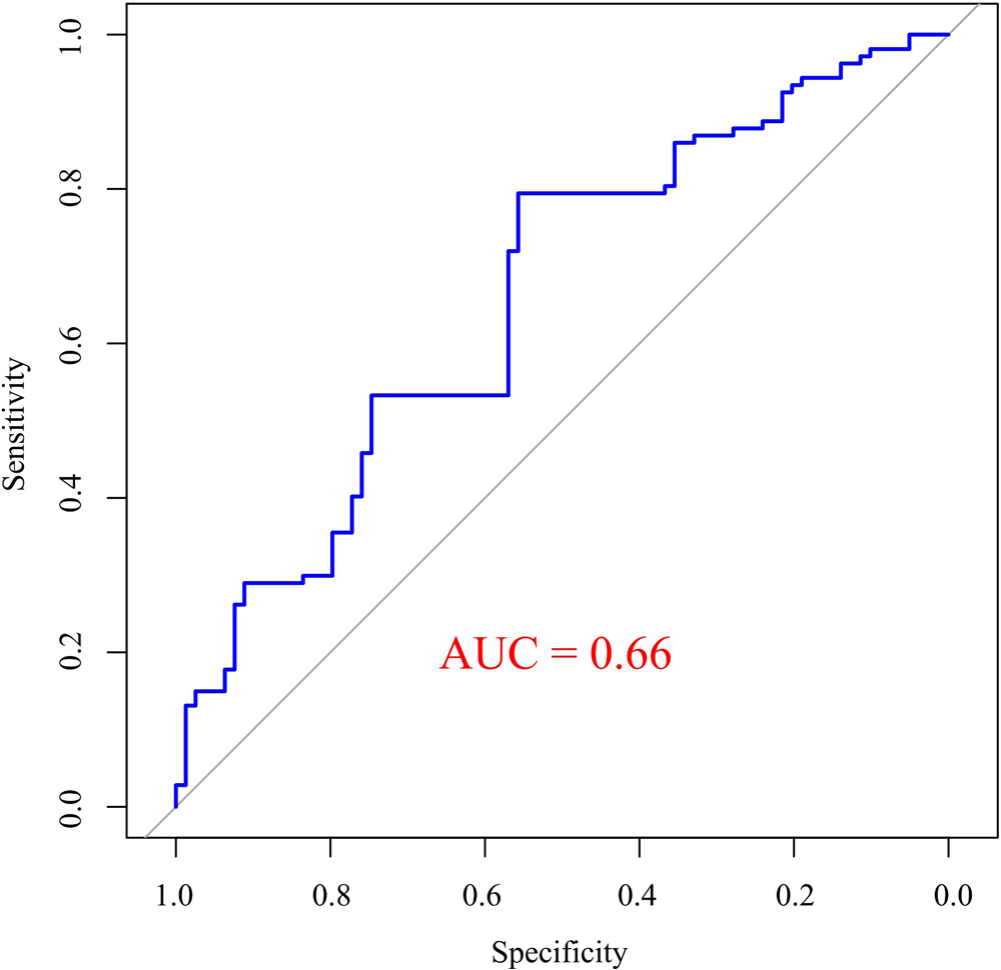
ROC analysis of the ZJU index’s predictive ability for CAS. AUC = area under the curve, CAS = carotid atherosclerosis, ROC = receiver operating characteristic.

Receiver operating characteristic analysis was performed to assess the predictive value of the ZJU index for CAS (Fig. 2). The results indicated that the area under the curve for the ZJU index in predicting CAS was 0.66 (95% CI: 0.63–0.70, *P* <.001).

### 3.4. Sensitivity analyses

We evaluated the stability of the association through sensitivity analysis (Table 3). According to the Chinese adult BMI classification standards, a BMI ≥28 kg/m^2^ is defined as obesity.^[13]^ After excluding 306 participants with baseline obesity, the association between the ZJU index and CAS remained unchanged. In the fully adjusted model, when the ZJU index was treated as a continuous variable, each 1 standard deviation increase was associated with a higher likelihood of CAS (OR = 1.09, 95% CI: 1.04–1.15). When the ZJU index was treated as a categorical variable, participants in the highest tertile of the ZJU index (ZJU index ≥42.06) had a higher likelihood of CAS compared to those in the lowest tertile (ZJU index ≤38.40).

**Table 3:** Sensitivity analysis of the association between ZJU index and CAS.

	Model 1 OR (95% CI), *P*-value	*P* for trend	Model 2 OR (95% CI), *P*-value	*P* for trend
CAS	–	<.001	–	<.001
ZJU index (per SD increase)	1.15 (1.10–1.20), <.001	–	1.09 (1.04–1.15), .001	–
ZJU index group				
Quartile 1: ≤ 38.40)	1.0 (Ref)	–	1.0 (Ref)	–
Quartile 2: 38.40–42.06	2.04 (1.36–3.08), <.001	–	1.54 (0.97–2.44), .067	–
Quartile 3: ≥ 42.06	4.28 (2.74–6.80), <.001	–	2.03 (1.15–3.62), .015	–

Model 1 adjusted for none; Model 2 adjusted for BMI, visceral fat area, WBC, HbA1c, TC, and HDL-C.

BMI = body mass index, CAS = carotid atherosclerosis, CI = confidence interval, HbA1c = hemoglobin A1c, HDL-C = high-density lipoprotein cholesterol, OR = odds ratios, TC = total cholesterol, WBC = white blood cell count.

## 4. Discussion

This study explored the relationship between the ZJU index and the occurrence of CAS in patients with T2DM and evaluated the predictive value of the ZJU index for CAS. The results showed that a higher ZJU index was closely associated with the occurrence of CAS.

Insulin resistance is a key mechanism regulating cellular metabolism and is closely related to the progression of metabolic disorders.^[14]^ Studies have shown that insulin resistance leads to early atherosclerosis, dyslipidemia, hyperglycemia, and hypertension.^[15]^ Moreover, even after correcting for hypertension, dyslipidemia, and blood glucose levels, the risk of cardiovascular disease remains elevated in patients with T2DM, primarily due to insulin resistance-induced atherosclerosis.^[16]^ Surrogate assessments of insulin action have been widely applied to study the relationship between IR and various clinical syndromes, leading to the development of multiple IR surrogate indices. For instance, the triglyceride-glucose (TyG) index, calculated based on fasting TG and blood glucose levels, has been proposed as a marker of insulin resistance.^[17]^ A longitudinal study showed that individuals with a higher TyG index had a significantly increased risk of subclinical atherosclerosis.^[18]^ Additionally, a cohort study involving 6028 participants indicated that elevated TyG index levels were associated with an increased risk of arterial stiffness.^[19]^ Recently, the metabolic insulin resistance score (METS-IR), a new insulin sensitivity screening system, has been shown to effectively identify individuals at high risk for IR-related pathological changes. The METS-IR calculation includes not only FBG and TG but also BMI and HDL-C. Studies have shown that METS-IR is significantly correlated with liver and pancreatic fat content, and ectopic fat accumulation in muscles and the liver is considered a major mechanism for the development of IR.^[20,21]^ Therefore, METS-IR is not only a tool for assessing insulin resistance but also an effective tool for predicting the risk of metabolic diseases.^[22]^

The ZJU index, in addition to combining BMI, FBG, and TG, also includes information on ALT and AST, reflecting liver metabolic abnormalities. In a cohort study of 28,729 Chinese adults initially free of nonalcoholic fatty liver disease (NAFLD), the ZJU index was significantly associated with the risk of developing NAFLD, with increasing ZJU index quartiles corresponding to higher risk ratios, with females at 4.87 and males at 6.23.^[23]^ However, existing studies have mainly focused on the correlation between the ZJU index and the incidence of NAFLD, with limited information on its relationship with atherosclerosis. The results of this study show that the ZJU index is an independent factor associated with CAS. Furthermore, a study involving 3329 Chinese adults showed a significant correlation between the ZJU index and insulin resistance.^[24]^

As a comprehensive metabolic health indicator, the clinical utility of the ZJU index has been validated. The ZJU index not only effectively reflects various metabolic abnormalities in the body but also serves as a simple and economical tool for early screening of metabolic disorders, especially in predicting the risk of atherosclerosis in diabetes patients. Since the ZJU index includes commonly available and easy-to-obtain clinical parameters, it has high operability in daily clinical practice. It helps clinicians to identify high-risk patients and take appropriate intervention measures without the need for complex or expensive tests. As the clinical applications of the ZJU index continue to expand, it provides an important reference for the early identification and intervention of atherosclerosis.

This study has several limitations. First, as a cross-sectional study, it is important to note that causality cannot be established. Second, although several potential confounders were adjusted for, there may still be unmeasured confounding factors that could influence the observed association. Third, the study lacked data on diabetes treatment medications and insulin levels, which may have affected the results. Fourth, the predictive ability of the ZJU index was not compared with common insulin resistance surrogate indices, such as the TyG index and METS-IR. Fifth, the study was conducted during the COVID-19 pandemic, which may have introduced selection bias due to the higher hospitalization rate among patients with high BMI, potentially affecting the sample composition. Finally, the generalizability of the findings is limited, as the sample was specific to patients from Cangzhou People’s Hospital. Further studies involving more diverse populations are needed to validate these results. To address these limitations, future prospective studies should expand the sample size and data range to further validate our findings.

## 5. Conclusion

The ZJU index may serve as a potential predictive indicator of CAS in patients with T2DM.

## Author contributions

**Data curation:** Xiao Yu, Jiaming Shen, Mengmeng Wang.

**Formal analysis:** Xiao Yu, Jiaming Shen, Mengmeng Wang.

**Funding acquisition:** Yazhao Sun.

**Methodology:** Xiao Yu, Jiaming Shen, Mengmeng Wang.

**Writing – original draft:** Xifeng Sun, Yazhao Sun.

**Writing – review & editing:** Xifeng Sun, Xiao Yu, Jiaming Shen, Mengmeng Wang, Yazhao Sun.
